# Benefits and challenges of multi-level learner rural general practices – an interview study with learners, staff and patients

**DOI:** 10.1186/1472-6920-14-234

**Published:** 2014-10-24

**Authors:** Tracy Morrison, James Brown, Melanie Bryant, Debra Nestel

**Affiliations:** Victoria University, Osteopathic discipline, College of Health and Biomedicine, Melbourne, Victoria Australia; Southern General Practice Training, Churchill, Victoria Australia; Swinburne University of Technology, Swinburne Business School, Hawthorn, Victoria, Australia; Monash University, School of Rural Health, HeathPEER, Faculty of Medicine, Nursing and Health Sciences, Clayton, Victoria Australia

**Keywords:** Multi-level learners, Vertical integration, Rural general practice, Education, Registrars, Interns, Medical students

## Abstract

**Background:**

General practices vary in the provision of training and education. Some practices have training as a major focus with the presence of multi-level learners and others host single learner groups or none at all. This study investigates the educational benefits and challenges associated with ‘multi-level learner’ practices.

**Methods:**

This paper comprised three case studies of rural general practices with multiple levels of learners. Qualitative data were collected from 29 interviews with learners (n = 12), staff (n = 12) and patients (n = 5). Interviews were initially analyzed using open and axial coding and thematic analysis.

**Results:**

Thematic analysis showed ‘multi-level learning’ in general practices has benefits and challenges to learners and the practice. Learner benefits included knowledge exchange, the opportunity for vertical peer learning, a positive learning environment and the development of a supportive network. The presence of multi-level learners promoted sharing of knowledge with all staff, a sense of community, an increase in patient services and enthused supervisors. Challenges for learners included perception of decreased access to supervisors, anxiety with peer observation, reduced access to patient presentations and patient reluctance to be seen by a learner. Practice challenges were administration requirements, high learner turnover, infrastructure requirements and the requirement for supervisors to cater to a range of learner level needs.

**Conclusions:**

The presence of medical students, interns and registrars in general practice has educational benefits to the learners extending to the other stakeholders (staff and patients). Multi-level learners present challenges to the learners and the practice by increasing pressures on resources, staff (administrative and supervisors) and infrastructure.

## Background

General practices vary in provision of training and education. While some have training as a major focus with multiple levels of learners (medical students, interns and registrars), others host single-level learner groups or none at all. This study explores the educational benefits and challenges of multi-level learner general practices in a rural setting. We define multi-level learner general practices as providing education across undergraduate (medical students), pre-vocational (interns) and vocational (registrars) programs.

Much literature is available exploring education in general practice settings including papers investigating various aspects of having a single learner group in the practice e.g. medical students [1–7] or registrars [8–11]. Specific studies conducted in the general practice setting with multi-level learner presence include an exploration of; challenges of teaching in general practice [12], the capacity with which practices may increase teaching load [13], financial considerations of learners in the practice [14, 15], key elements of a primary care teaching practice [16], benefits and risks of shared learning models [17], facilitating factors and barriers to shared learning [18] and, how clinical learning occurs [19].

The presence of multi-level learners can enable *vertical integration, which* is a topic of increasing interest in literature. Vertically integrated training comprises of "continuous educational pathways, all stages in GP education, supporting the continuing educational/professional development needs of learners at each stage and effective curriculum planning and delivery" [20]. Of the seventeen Regional Training Providers (RTPs) of general practice education in Australia, eleven identified they have undertaken vertical integration initiatives [21]. There are many benefits of vertical integration/multi-level learners in general practices including a reduction in supervision load [22] and increase in supervisor satisfaction [17], while creating peer learning opportunities, which allow further for focus on high quality teaching activities [23] and opportunities for positive changes within the practice; greater financial efficient and a more sustainable practice [17]. However supporting multi-level learners and vertical integration in general practices is challenging for learners, supervisors and the practice. Learners at varying levels of their education have different needs [17] and additional resources are required, such as financial support and more infrastructure [24]. Varying levels of engagement and teaching skills of supervisors/registrars and evaluating the quality of teaching have also been proposed as challenges [24].

We extend the work identifying benefits and challenges of multi-level learners and vertical integration. This paper builds on similar work by Ahern and van der Mortel and colleagues [17, 18] identifying educational benefits and risks of shared learning models in general practices – that is, practices with the three learner groups present. Our study explored educational benefits and challenges of multi-level learners in general practices as well as benefits and challenges to the practice as a medical service organization. We included patients as participants as we were interested to investigate to what extent the recipients of the medical service were directly or indirectly affected. Our research design is similar to the Pearson and Lucas [16, 19] who adopted a single case study design to explore key elements of general practice and clinical learning in multi-level learner settings. We offer data from a collective case study approach [25] with three practices (cases) and sought feedback from multiple stakeholder perspectives (learners, staff and patients) and focused on rural general practices.

The research questions are:

1. What are the benefits of multi-level learner practices?
2. What are the challenges of multi-level learner practices?

## Methods

### Researchers

The research involved a qualitative study conducted from an interpretivist approach [26]. This enabled the research team to focus on the experiences of individual participants and how they interpret their surroundings through interactions with others. It also acknowledges researcher reflexivity in which our own backgrounds and experiences influence the way we approach and analyze the data [27]. The research team consisted of an allied health professional and clinical educator (TM), a general practitioner and medical educator (JB), an organizational behavioral theorist (MB) and an educationalist (DN). The data collection and analysis involved all members of the research team. Several rounds of discussions were undertaken to ensure that the interpretation of the data reflected the interviewee’s responses and that the research questions were addressed. This team approach was used to enhance the credibility and trustworthiness of the findings as appropriate for an interpretivist study [28].

### Setting and participants

The study involved three multi-level learner general practices located in rural areas in the state of Victoria, Australia. The practices were selected because of their strong engagement in educational activity with multiple levels of learners and stated vertical integration. Learners (medical students, interns and registrars), staff (supervisors, nurses, practice managers and receptionists) and patients were purposively sampled so that each stakeholder group was represented. Actual numbers of participants per practice were determined by the availability of learners and staff in the practice at the time of the study. Patient numbers were determined by the number of patients who volunteered for the study. The practice manager informed participants of the research and provided a copy of the explanatory statement. Participants indicated their willingness to participate by contacting the practice manager and scheduling an interview time. Participants were provided the first authors contact details for any points of clarification before the interview. Of those learners, staff and patients invited to participate, all consented to the study through signing a consent form at the time of data collection. None of the participants were provided with inducements to take part in the research.

### Data collection and analysis

Data was collected from 29 participants using semi-structured interviews over a three months period from January to March in 2012 within each of the practices by TM and MB. A topic guide was developed for each stakeholder group (see Appendix 1). This method of data collection enabled exploration of relevant issues in each practice in context. Interviews ranged from twenty to sixty minutes (average 33), were audio taped and transcribed verbatim. The data were analyzed in several stages. The first stage involved four members of the research team independently coding transcripts using a combination of inductive open and axial coding. This enabled the development of code labels and exploration of relationships between codes, which formed the basis of a common coding template [29]. This template was then used as a guide in the second stage of analysis in which transcripts were analyzed by practice thematically from which a series of key and sub-themes emerged inductively. A cross-case analysis was then undertaken to compare the themes across each practice as an interpretivist approach was used to guide the research we do not seek to generalize the findings into different settings. However, we argue that our findings can certainly be used to inform other studies.

Ethics approval was obtained by the Monash University Human Research Ethics Committee – project number CF11/3006 – 2011001694. The three practices involved provided permission letters indicating their approval to being involved in the study.

## Results

### Overview of practices

Although each practice appeared to have its own identity, there were many similarities. The similarities included, strong commitment to education particularly expressed in the passion of the practice supervisors, several administration staff (more than two, reflecting a larger practice), supportive practice community and patient awareness of the learners within the practice. Notable differences between the practices were the total number of learners (p1 = 5, p2 = 3, p3 = 5), supervisors (p1 = 3, p2 = 2, p3 = 1) and the model of supervision (practice 3 did not consult while supervising while practice 1 and 2 parallel consulted).

### Participants

The overall sample included 29 participants (12 learners, 12 staff and five patients) and the number and position of participants per practice is summarized in Table 1.

**Table 1:** **Number of participants per stakeholder group**

	Learners			Staff				Patients
Practice	***Medical students***	***Interns***	***Registrars***	***Supervisors***	***Nurses***	***Receptionist***	***Practice manager***	
1	2	1	2	2	1	1	1	2
2	1	1	0	1	1	1	1	1
3	2	2	1	1	1	0	1	2
Total	5	4	3	4	3	2	3	5

### Themes and sub-themes

Tables 2 and 3 summarize two groups of themes: 1) benefits to learners and the practice and 2) challenges to learners and the practice. We define learner benefits/challenges as factors that impact directly on the learners’ educational development. Practice benefits/challenges are factors that impact on the practice environment or the people who interact with the learners as they are undertaking their placement within the clinic. Illustrative quotes of the themes and sub-themes from the three participant groups are provided in Tables 4, 5, 6 and 7.

**Table 2:** **Themes and subthemes on benefits in multi-level practices**

Learners	Practice
Knowledge exchange	Knowledge exchange with others
Vertical peer learning	Sense of community
Positive learning environment	More patient services
Supportive network for learners	Supervisor satisfaction

**Table 3:** **Themes and subthemes on challenges in multi-level practices**

Learners	Practice
Perception of decreased access to supervisor	Administration
Feeling anxious	Learner turnover
Competition for cases	Infrastructure
Patient reluctance	Supervisor workload

**Table 4:** **Data display of verbatim statements in themes and sub-themes** ***‘Benefits to learners’***

Sub-theme	Learners	Staff	Patient
Knowledge exchange	*I think having clinical experiences to reflect on and cases to present is the most helpful for me as a student. And when you are learning with medical students at the same level you have the same level of clinical experiences, and you don’t have as many things to reflect on. But the registrars have those experiences. And they (registrars) get a bit further away from the pathophysiology. So that sort of less clinical knowledge is something that’s more fresh to me; so they get kind of reminders about that from me in teaching sessions. (Male medical student#1 at P2)*	*And we had multi-level teaching, so it was just a wonderful forum for sharing knowledge and for bouncing ideas and for learning and for relationship building. (Male supervisor#1 at P2)*	
Vertical peer learning	*They (the registrars) get a bit further away from the pathophysiology. So less clinically relevant knowledge is something that’s fresh to me because I have just learned it, so they get kind of reminders about that from my input. (Male medical student#1 at P2). really like having the student because we did the same course and she’s a couple of years below me, so I’m just delighted to be able to teach someone something. (Female intern#1 at P2)*	*The registrars taught the interns, the registrars and interns taught the medical students. In fact, that’s where most of the teaching happened for the medical students, in a lot of sense, to build them up to the exams, and also when the registrars were having exams they taught each other. (Male supervisor#1 at P2)*	
Positive learning environment	*But it’s nice to have them (students) around and I think if we’re just out of university so we’re still sort of mucking around and still have that mentality, so it’s kind of nice to have students around.(Female intern#1 at P3). Because you have to feel safe to [ask questions] and in the learning sessions that I talked about, every Tuesday, we can present things that that have come up as a point of interest, and so that keeps it current for the learners. (Female intern#2 at P3)*	*I listen, you can hear them, they’re laughing, they’re enjoying it, they’re challenging each other and they jump on the computer there, and they will bring it up on line. From that perspective they’re positive about what they’re doing, it’s not a threatening environment. It’s not oh jeez, we’ve got to go to the teaching session. (Male practice manager#1 at P2)*	
Supportive network for learners	*When if there has been a difficult patient that’s frustrated you about something, you can vent (to the other learners), and I think everyone is aware that the content of what’s coming out of your mouth, when it does sound harsh, is just a vent and its actually affected me and I just need to say something, just to get it out there and have someone just sort of be there. (Female intern#1 at P1)*	*So that they (the learners) form this nucleus of like people, may be form friendships and that sort of stuff, which helps out; it helps out not only between the clinics but also if they’re working in the hospital. (Female receptionist#1 at P3)*

**Table 5:** **Data display of verbatim statements in theme and sub-themes** ***‘Benefits to the practice’***

Subtheme	Learners	Staff	Patient
Knowledge exchange with others	*General practitioners often say that they learn a lot off the students too, because students actually have an opportunity to read the most current literature. So they will update the doctors, rather than the doctors trying to fit that in their busy day. (Female medical student#2 at P1)*	*And I know the staff here enjoy it because there’s young people with new ideas and new ways of looking at things, or they will challenge; which is good for the older person because it means that they’re learning, and they’re refreshing themselves. (Male practice manager#1 at P2). There’s more interaction (with learners present). But you learn a lot more. I think you listen, and you, because there is more explaining, I myself find that I learn a lot more too because the doctors are explaining to the interns, and I think it’s a learning (curve) here for all of us (Female nurse#1 at P1)*	*(Having the learners) keeps the doctors up to date with current stuff, because I have heard it. You’d be talking, and one of the interns would go, ‘oh yeah, but they have this new drug’ etc. So, both feed off each other. You know what I mean? Like, the old feed off the new and that keeps our older doctors younger and makes our younger ones a bit smarter.’ (Female patient#1 at P1)*
Sense of community	*So we have our own little team going and we do a lot of, we have a lot of communication between all of us. And I live with one of the interns. So I see her every morning and night and we usually have a bit of a debrief about the day (Female intern#2 at P3).*	*They (learners) make the overall organisation vibrant and active and moving forward; we have to do this (move forward) to meet an educational standard meaning that we’re all having to meet a whole lot of personal standards, and that would be, to my mind, the biggest benefit of the whole thing. Although it adds to general satisfaction for everyone too, I think. (Male supervisor#2 at P1)*	
More patient services	*And I think as a practice we seem to be able to accommodate more people for emergencies now we’ve got that extra person to do those things (Female registrar#1 at P1)*	*It just helped, that extra person to help with patient load and that sort of thing; and when at first we used to get, the interns would help us quite a bit in the treatment room, if a patient needed a script or something we would get them to write it up, or any triages. (Female Nurse#1 at P2)*	*Before they started (having) the interns here it was hard to get an appointment there for… eighteen months. (Female patient#2 and P3)*
Supervisor satisfaction	*I know that they (supervisors) look forward to their teaching sessions, because they get something out it too, you know, and you, you know, well I dare say if you’ve been in that environment where you’ve been teaching or educating somebody you get a lot of enjoyment out of seeing them grow. (Female medical student#2 at P3)*	*Well, I do it for my own good because I enjoy teaching, I enjoy the learning I achieve by the accumulation of knowledge from all the learners. (Male supervisor#1 at P2)*	*No, I think it brings a lot to them, as well. Because they’re teaching something that, obviously, they love. (Male patient#2 at P1)*

**Table 6:** **Data display of verbatim statements in theme and sub-themes** ***‘Challenges to learners’***

Subtheme	Learners	Staff	Patient
Supervisor time divided	*Sometimes, if there weren’t enough supervisors and because the intern also had to be supervised so they were basically seeing a patient and then the GP had to go in afterwards and see the patient as well; so in that sense sometimes it could be a bit busy especially if our supervisor’s supervising us, both myself and the intern. So a bit of time is wasted. (Female medical student#2 at P1)*	*There’s probably about an hour or so of interpersonal interaction added on. We must have something like six or seven learners in the building over the course of a week, and if each one of them is having a bad week, that adds up to a significant amount of time. (Male supervisor#1 at P1)*	
Feeling anxious	*But for the junior doctor it is quite nerve wracking having someone watching you, It is always nerve wracking having the medical student watching. (Female intern#1 at P2)*		
Competition for access to patients	*All this talk about getting more junior doctors out in to clinics to learn more; they’re not going to learn anything if you don’t have the patients to back them up. (Female intern#1 at P3)*		
Patient reluctance	*I’ve received annoyances from patients was patients that said ‘oh I’m seeing you today, I was hoping to see (the senior doctor). (Female intern#1 at P1)*	*Although the patients tended not to want to see the Interns because they hadn’t had any relationship building. (Male supervisor#1 at P2)*	*I don’t see a negative there but my husband doesn’t like it; he wants to see the same doctors, not the learners (Female patient#1 at P1)*

**Table 7:** **Data display of verbatim statements in theme and sub-themes** ***‘Challenges to the practice’***

Subtheme	Learners	Staff	Patient
Administration	*I suppose there’s a lot of organisation that needs to happen. They’ve got the practice manager here who works really hard organizing accommodation and paperwork, and I guess the interns where you rotate through here every ten weeks; so, you know, every ten weeks you’ve got someone turning up who’s new, doesn’t know where anything is, and you have to start all over again. (Female intern#1 at P2)*	*There’s, each level of learner has its own bureaucratic requirements, bureaucratic/ legitimate educational requirements too, you know, feedback, reports, mid term assessments, you know, teaching and consultation records, some of which can be done at the admin level but not all of it can. (Male supervisor#2 at P1)*	
Learner turnover	*Supervisors like to teach and they must like to share, maybe because they like to see people grow…but on the other hand…they don’t get to set up the relationships to last for a long time. (Female intern#1 at P3)*	*One of the disadvantages is, when you do get a fantastic intern…they’re only here for ten weeks and not only would we like them to stay, they’d love to stay as well. (Practice manager#1 at P3). It is problematic because what happens is that someone comes into the community, we bend over backwards to make them feel welcome and they join in to more or less an extent depending on their personality, and then they leave after six months or a year. (Female practice manager#1 at P1)*	
Infrastructure		*Well, it puts a strain on the infrastructure. Each learner in a consulting room is going to see less patients using that consulting space than a fully experienced GP, so, in terms of infrastructure, in terms of seeing patients, you’d do better without having a medical student there and finding a full time doctor. (Male supervisor#2 at P1)*	
Supervisor workload	*I think the challenge has got to be for the supervisor to keep stretching the learners and to know what they should know and to pitch their questions. So I think it’s important. I don’t think the challenge is so much for the learners. I think it must be more for the supervisor… So I think that’s a bigger challenge because amongst themselves they know what they can do and stretch each other all the time, (Female intern#2 at P3)*	*You have to work through the issues and basically be there for the each group of learner. (Male supervisor#1 at P2)*	

### Benefits to learners

Learner benefits included sharing knowledge and experience, vertical peer learning, a positive learning environment and the presence of a supportive network.

Learners across the practices reported that each level of learner has different knowledge and experience based on their stage of medical training. Registrars were seen to have greater clinical experience which medical students and interns valued. Interns and registrars reported that medical students offer a different type of knowledge base, as their studies at medical school are largely the theoretical aspects medicine.

Each practice offered structured teaching sessions where a supervisor facilitates learning with medical students, interns and registrars. This was a forum for sharing knowledge and experience.

Some registrars valued medical students and interns observing their consultations as the teaching opportunity challenged their practice. Registrars were motivated to reflect on their own practice and this was seen to aid their learning. The four interns across the practices all discussed the benefit of sharing their experience of medical school with medical students and valued teaching a junior. Although the medical students did not see themselves as teachers, the interns and registrars stated they learned from the student contributions in teaching sessions.

Learners and staff reported that the presence of several learners creates an encouraging and positive learning environment. Junior learners (medical students and interns) valued having a peer who was close to their stage of training. In this setting, they felt more comfortable to ask questions of the supervisor and of each other. Registrars, being the most senior learner gained encouragement from sharing their experiences with their junior colleagues. Several practice staff reported that the presence of multi-level learners strongly supported a culture of learning, which encourages the learners to feel ‘at home’ within the positive learning and practice environment.

Learners, staff and patients discussed the benefit to learners in developing a supportive network. As these practices were rurally located, it was common for learners to be away from family and friends. The three practice managers highlighted the need to ‘welcome’ the learners into the community, irrespective of the length of the rotation. Learners found support through this supportive network and from social encounters outside the clinic. Supervisors and practice managers viewed support as a high priority for learners within their practice and implemented measures to create it. Examples of this were orientation processes, accommodation in close proximity to other learners and social functions.

### Benefits to the practice

The sub-themes were knowledge exchange with others, sense of community, increase in patient services and supervisor satisfaction.

While knowledge exchange occurred between learners and supervisors, others in the practice also benefited. Nursing staff reported learning as a consequence of the presence of multi-level learners because they observe more interaction and discussion in the clinic. For example, the doctors explain the treatment and care of the patient to learners, which often is a learning experience for the nurse too. An illustrative quote from a nurse in one of the practices can be viewed in Table 5.

Patients and staff of these practices also identified that learners impact on the sense of community by bringing vibrancy, enthusiasm and new perspectives. One feature of a rural community is a familiarity between the people who live there. It was common for patients and staff to know each other well. Learners often reported feeling extremely welcome and spoke of strong learning cultures.

The introduction of interns to practices contributes to the health service delivery as they carry a caseload themselves. Staff and patients at all three practices appreciated the availability of interns for increased appointments. It was common for staff doctors at the three practices to be fully booked a week in advance. Interns were available to see patients who booked at short notice.

Learners, practice staff and patients indicated they see a high level of satisfaction in the practice supervisors. The supervisors identified self-satisfaction of teaching in multi-level learner practices, enjoying gaining knowledge from learners and remaining involved with patients being consulted by learners.

### Challenges to learners

Learner challenges included perceived decreased learner access to the supervisor, increased anxiety among some learners, reduced exposure to patients with the caseload distributed between a number of learners and patient reluctance to see junior learners.

The presence of more learners requires greater supervisory responsibilities. Although the number of supervisors varied between the practices from one to three, there were consistent comments from learners across each level that the access to the supervisor had to be shared between learners. Supervisors often reported there was time pressure involved in supervision, but did not explicitly indicate it was heightened due to multi-level learner presence.

While some learners (particularly registrars) valued the presence of learners as observers, two of the interns spoke of an increased feeling of anxiety when the medical student was observing their consultations. The same interns embraced the opportunity to share knowledge and teach the students but this was generally outside the consultation room. Medical students did not have interns or registrars observe their consultations.

Practices with a greater number of learners can have reduced learning opportunities due to decreased access to patients for each learner. This was most obvious for the medical students who said an intern would have ‘priority’ to certain learning opportunities (e.g. suturing and wound care). Registrars and interns were generally satisfied with their access to patients although a registrar considered that s/he saw less acute care patients than the intern due to the way patients were booked.

Patients expressed some reluctance to see learners. Registrars did not experience this but interns and medical students sensed that some patients were reluctant to fully engage in their consultation because of their junior level. Staff also reported that some patients are unhappy when required to repeat their medical history to a new learner.

### Challenges to the practice

Practice challenges were administration, learner turnover, infrastructure and disparate learner needs. Different administrative requirements are associated with different learner levels with implications for workload, particularly on practice managers and supervisors. There are different educational institutions supporting each learner type. For medical students, the practice liaises with the university, for interns it is the Post Graduate Medical Council and the Regional Training Provider (RTP), and for the registrars it is the RTP. Billing arrangements are different for registrar and interns. Registrars bill patients through Medicare under the registrar’s provider number. The interns’ patients are billed under the supervising doctor’s provider number. All learners require orientation to the clinic. They also require entering into the patient management system so appointments can be made. There are also reporting requirements for each learner.

Learners in general practices operate on a rotational basis of different lengths for each learner group. Medical students placements normally extend the entire year. Intern placements are ten weeks. Registrar placements are normally six months. The turnover of learners in multi-level practices can be challenging, particularly as staff and patients may form significant relationships with the learner. The high turnover can be disruptive to the clinic morale.

Practice managers, supervisors and receptionists across practices noted learners require their own consultation room, which limits available rooms for GPs. Two practices have extended their facilities in the last ten years to cater for growing number of learners and need for learner-dedicated teaching space. The third practice has made modifications to existing facilities to provide suitable space for learners.

## Discussion

These results demonstrate that hosting multi-level learners offers both clear educational benefits and also challenges to all learner groups, staff and patients at a multi-level learner practice. A clear educational benefit is the opportunity for vertical integration through near peer learning. This also contributes to reducing the load on supervisors, one of the previously reported benefits of vertical integration [22]. One of the practice challenges identified in this study is the necessity for the supervisor to be aware of each learner group’s curriculum and some supervisors see this as additional work. Although registrars relieve supervisors of some of their teaching burden by teaching interns and medical students, supervisors are still required to maintain an involvement in the education of all learners in the practice. This finding is related to previous work which identified a risk of shared learning in general practice perceived by learners is meeting individual learning needs [17]. These results highlight the importance of supervisors feeling adequately resourced and prepared to cater for individual learner needs to ensure learners have a tailored and valuable learning experience.

Participants clearly identified multi-level learner presence facilitated knowledge exchange which has been proposed as a benefit of multi-level learner presence [23]. Our data showed knowledge exchange occurs at two levels. Firstly, the learners have the ability to share knowledge with each other, which we see as a learner benefit, and secondly, knowledge is shared between the learners and staff which is a benefit to the although it is likely it also benefits the learners by creating a culture of learning. Supervisors have reported they learn with learners in the practice [17] and our data set showed supervisors and other practice staff (e.g. nurse) also learn. There is also potential benefit to the patients as observed in our data, which is a new finding.

Collaborative or cooperative learning models in higher education are manifested through peer learning [30] and are well documented in the medical education literature [31–36], especially in medical school. There are fewer references based on studies of work-place based learning utilizing peer assisted learning. Vertical peer learning, where near peers learn from each other is a strong feature of our data. Connecting near peers by sharing theoretical and practical concepts seemed to offer benefits to all. Further, near peers often offer something that supervisors are unable to offer since they are too far from the experiences of novices [37].

Other benefits of multi-level learners identified by Ahern and colleagues [17] are the opportunity for more patient services to be offered and a feeling of supervisor satisfaction. Our analysis found these two benefits were reported by all stakeholder groups in our study supporting the previous work by offering diversity in perspectives. Van der Mortel and colleagues identified enabling, factors of shared learning in general practices [18] which need to be considered when aiming to maintain these benefits. The enabling factors identified were organizational/administrative, structural, resources and teaching and facilitation skills and these relate to our findings under the challenges to the practice theme. Administration was seen as a challenge in our data and can also be perceived as an enabling factor to shared learning. Structural considerations such as practice size, number of supervisors and space [18] relates to our infrastructure sub-theme where staff identified the need to ensure learners their own consulting room which can limit rooms available for GPs. Practices will need to expand their infrastructure to ensure benefit of offering more patient services is not compromised. Our study also found the rotational basis of learners throughout the practice can be challenging which has not been reported previously.

Glasgow and Trumble [24] proposed the following challenges to vertical integration; practices being resistant to change, limited infrastructure and time, and lack of financial support for program development. Our results support two aspects of these challenges. First, we found an increase in the administration (and therefore time) required by staff and second, we found the need for suitable infrastructure (more consulting rooms). The three practices in this study did not however exhibit a resistance to change. This study adds to Glasgow and Trumble’s work by identifying specific challenges to the learners. The perception of supervisor’s time being divided between levels of learners may have an impact on the quality of educational experiences. However, learners also identified several benefits of undertaking their placements together. The question of whether learner benefits (resulting from their close interaction) outweigh the challenges cannot be answered by this study.

### Strengths and limitations

The major strength of this study is the multiple stakeholder approach and opportunities for cross case comparison of the three practices. However, the practices were different in their number of learners, supervisors and approach and we do not know if these differences influenced the perspectives of the participants as it was not a specific aim of our study and generalizability of findings to other practices and clinical settings is unlikely. The interview based study yielded a large data set and saturation was reached but we do not know if the practices are representative of rural general practices in Victoria. Although we believed the inclusion of patients was important to deepen insights, data from patients did not make significant contributions to each theme. An additional limitation relates to the learner participant group which included five medical students and interns but only three registrars. This may result in under representation of registrars’ perspectives although saturation was achieved.

## Conclusion

The benefits of learners undertaking their training together extend to the learners, supervisors, the practice and patients. Important considerations for multi-level learner practices include available supervisors, sufficient caseload, and administration support. This study adds to the previous work on multi-level learners in general practices by supporting previous studies results of benefits and challenges and offering new findings, particularly those we termed ‘benefits to the practice’ with sub-themes of; knowledge exchange (with staff and patients), sense of community (within the practice), increase in service provided (more appointments available) and supervisor satisfaction.

## Appendix 1: Topic guides for individual interviews

### Patients

General questions

- Why do you visit this medical practice?
- How long have you been coming to this practice?
- Have you noticed any changes over this time?
- What do you like about this practice?
- Can you describe any aspects you don’t like?
- Can you describe a consultation you have had at this practice?
- Who did you see?
- Who did you talk to?
- What happened during the consultation?
- What are your expectations when you visit this practice?
- To what extent are these expectations met?
- Can you describe a situation when your expectations were met?
- What factors do you think contributed to your expectations being met?
- Can you describe a situation when your expectations were not met?
- What factors do you think contributed to your expectations not being met?
- What have you seen change at the practice in your experience as a patient?
- How have these changes affected your experience as a patient?
- Have you been ‘seen’ by students when you have been in this practice? If so, how was it?
- What about junior doctors? Are you aware if you have consulted junior doctors? If so, how was it?
- And what about doctors who are training to be GPs? Are you aware if you have consulted these doctors? If so, how was that?

There may be advantages and disadvantages having learners in the practice.

18. What do you think the advantages are?
19. What are the disadvantages?
20. Overall, what do you think about having learners in the practice?

Sometimes, general practices conduct research – like this project.

21. Are you aware of having participated in research projects? If yes, can you tell me what it was about? What did you think about it?
22. How important do you think it is that the doctors in your practice undertake research?
23. Is there anything else you would like to share?

### Practice staff

1. What is your role within this practice?
2. To what extent are you involved with the registrars/interns/medical students within the practice?
3. What are the advantages of having the registrars/interns/medical students within the general practice?
4. What are the disadvantages?
5. How do you think the presence of the learners influence the practice?

Feel free to comment on the:

- Atmosphere
- Service delivery (care of patients)
- Financial influence
- Supervisors/treating general practitioners
- What would the practice look like without learners?
- Benefits?
- Costs?
- How would you define an Academic General Practice?
- How would the practice be different if there was only one level of learner (academic practices only)
- Benefits?
- Costs?
- To what extent do the registrars/interns/medical students interact with each other (academic practices only)
- How would the practice be different if there were multiple level of learners (single level learner practices only)
- Benefits?
- Costs?
- What teaching resources and support for practices is provided?
- Who provided this?
- How effective are the current resourcing and support models?
- What works/doesn’t work?
- How could these be changed?

Organisational change

- Ideally, what would you like the practice to look like?
- What would need to change in the practice to become an academic practice?
- What are the current barriers to change?
- How could these barriers be overcome?
- What are the current strengths of the organisation that would enable an academic practice?
- How would practice strategies need to change to enable a rural academic practice?
- What would the implications for resourcing be, i.e., staffing levels; training; professional development; size of practice; current strategy?
- What resources are needed for change?
- What role do broader institutions play in ability to develop into an academic practice?
- What are the Issues surrounding resistance to change?

Research

- Can you comment on any research that has been conducted in this general practice?
- What was the aim of this research?
- Were you involved in the research?
- How did this research affect the practice?
- How important is it for general practices to be involved in research?
- What areas of research would you like to see general practices involved in?

Academic practices only

27. How has the practice changed in terms of day-to-day operation?
28. What were the key barriers to developing into an academic practice?
29. How did changing into an academic practice impact upon the organisational culture?

### Supervisors

1. What is your role within the general practice?
2. Why do you participate in student supervision?
3. What are the advantages of having the registrars/interns/medical students within the general practice?
4. What are the disadvantages?
5. How does the presence of the learners influence the practice?

Feel free to comment on the:

- Atmosphere
- Service delivery (care of patients)
- Financial influence
- Supervisors/treating general practitioners
- How would the practice be different if the learners were not present?
- Benefits?
- Costs?
- How would you define an Academic General Practice?
- How would the practice be different if there was only one level of learner? (academic practices only)
- Benefits?
- Costs?
- To what extent do the registrars/interns/medical students interact with each other? (academic practices only)
- How would the practice be different if there were multiple levels of learners? (single-level learner practices only)
- Benefits?
- Costs?
- What teaching resources and support for practices is provided?
- Who provided this?
- How effective are the current resourcing and support models?
- What works/doesn’t work?
- How could these be changed?

Organisational change

- Ideally, what would you like the practice to look like?
- What would need to change in the practice to become an academic practice?
- What are the current barriers to change?
- How could these barriers be overcome?
- What are the current strengths of the organisation that would enable an academic practice?
- How would practice strategies need to change to enable a rural academic practice?
- What would the implications for resourcing be, i.e., staffing levels; training; professional development; size of practice; current strategy?
- What resources are needed for change?
- What role do broader institutions play in ability to develop into an academic practice?
- What are the Issues surrounding resistance to change?

Research

- Can you comment on any research that has been conducted in this general practice?
- What was the aim of this research?
- Were you involved in the research?
- How did this research affect the practice?
- How important is it for general practices to be involved in research?
- What areas of research would you like to see general practices involved in?

Academic practices only

27. How has the practice changed in terms of day-to-day operation?
28. What were the key barriers to developing into an academic practice?
29. How did changing into an academic practice impact upon the organisational culture?

### Learners (Registrars, Interns, Medical students)

- What stage are you at in your medical training?
- How are you involved in the service delivery at the general practice?
- What do you like about being a registrar/intern/medical student within general practice?
- What do you find challenging?
- To what extent do you have interaction with other learners within the general practice?
- What facilitates this interaction?
- What limits it?
- What benefits do you experience through this interaction?
- Are there any negative aspects to this interaction?
- What are the advantages of having multi level learners within the practice? (academic practices only)
- What are the disadvantages?
- How does the presence of yourself and other learners influence the practice?

Feel free to comment on the:

- Atmosphere
- Service delivery (care of patients)
- Supervisors/treating general practitioners

Research

- Can you comment on any research that has been conducted in this general practice?
- What was the aim of this research?
- Were you involved in the research?
- How did this research affect the practice?
- How important is it for general practices to be involved in research?
- What areas of research would you like to see general practices involved in?

## Abbreviations

RTP: Regional training provider
GP: General practitioner.
